# Chandipura Virus Resurgence in India: Insights Into Diagnostic Tools, Antiviral Development, and Public Health Implications

**DOI:** 10.1155/ghe3/1015031

**Published:** 2025-04-21

**Authors:** Adewunmi Akingbola, Abiodun Adegbesan, Kolade Adegoke, Joel Chuku, Olajide Ojo, Petra Mariaria, Uthman Alao, Raolat Adenike Salami, Michael Oladunjoye

**Affiliations:** ^1^Department of Public Health and Primary Care, University of Cambridge, Old Schools, Trinity Lane, Cambridgeshire, Cambridge CB2 1TN, UK; ^2^African Cancer Institute, Department of Global Health, Stellenbosch University, Cape Town, South Africa; ^3^Faculty of Clinical Sciences, Obafemi Awolowo University, Ile-Ife, Nigeria; ^4^Department of Medicine, V. N. Karazin Kharkiv National University, Svobody Square, Kharkiv 61022, Ukraine; ^5^University of West England, Coldharbour Ln, Stoke Gifford, Bristol, UK; ^6^Department of Biomedical Laboratory Science, University of Ibadan, Ibadan, Nigeria; ^7^Afe Babalola University, Ado-Ekiti, Ekiti State, Nigeria; ^8^Department of Community Health, Lagos State University College of Medicine, Ikeja, Lagos, Nigeria

**Keywords:** antivirals, Chandipura virus, encephalitis, PCR, sandflies, vector-borne diseases

## Abstract

**Background:** Chandipura virus (CHPV) is an emerging rhabdovirus primarily affecting pediatric populations in India, causing acute encephalitis syndrome (AES) with high mortality rates. First identified in 1965, CHPV has resurfaced in several outbreaks, the most recent being in 2024, with significant public health implications. The virus is transmitted primarily by sandflies, particularly *Phlebotomus* spp., and has been associated with a rapid progression of symptoms, leading to severe neurological damage and death. Despite advances in diagnostic techniques, no specific antiviral treatment or licensed vaccine currently exists.

**Main Body of Abstract:** This manuscript reviews the latest findings on CHPV, focusing on diagnostic advancements, treatment strategies, and public health responses. Reverse transcription–polymerase chain reaction (RT-PCR) and enzyme-linked immunosorbent assay (ELISA) have emerged as vital tools for rapid and accurate diagnosis, enabling the identification of CHPV in clinical and environmental samples. Antiviral therapies, such as ribavirin and favipiravir, have shown promise in vitro and preclinical models, but human trials are lacking. Additionally, the virus's unique epidemiology, including its reliance on sandfly transmission, complicates control efforts, particularly in resource-limited settings. The 2024 outbreak, with a case fatality ratio of over 30%, highlights the urgent need for improved surveillance, vector control measures, and public health interventions to curb the spread of CHPV.

**Conclusion:** Despite considerable progress in diagnostics and experimental treatments, significant challenges remain in controlling CHPV outbreaks. The lack of specific antiviral therapies and vaccines continues to hinder effective management. Strengthened vector control strategies, advanced diagnostic infrastructure, and ongoing research into antiviral development are essential for mitigating the impact of CHPV in affected regions. International collaboration and sustained public health efforts will be crucial in preventing future outbreaks and reducing the disease burden.

## 1. Introduction

The Chandipura virus (CHPV) is a single-stranded RNA virus that was first isolated in 1965 from two adult patients suffering from a febrile illness in Chandipura village (Nagpur region) in India. The virus was identified during an outbreak of chikungunya and dengue viruses. Bhatt and Rodrigues, who first discovered and studied the virus, demonstrated a cytopathic effect in cells within 3 hours of administration of the purified strain of the CHPV into tissue culture [1]. However, beyond the discovery of the pathogen, its potential for epidemic transmission was not confirmed until isolates of the viral particle, viral RNA, or IgM antibodies were found in clinical samples of 51% of investigated cases during an outbreak of acute encephalitis of unknown origin in Andhra Pradesh, India, 2003, with a case fatality rate as high as 52.3% [2].

CHPV has since become a leading cause of encephalitis in the pediatric population with similar epidemic associations seen in the Gujarat region in 2004 with a case fatality ratio (CFR) as high as 75%. Outbreaks have also been recorded in Odisha in 2015 and among children in the Nagpur division of Maharashtra where 43.6% of infected children died from the disease with a classic clinical picture of high fever, vomiting, and diarrhea, along with eventual development of neurological symptoms, such as seizures, altered sensorium, and, in many cases, death [3–5]. Though the epicenter of the disease has been largely limited to central India, the virus has been isolated in a four-toed hedgehog in West Africa and sandflies in Senegal suggesting the possibility of wider incidence in the West African region. CHPV activity has also been reported from Sri Lanka after a study successfully detected antibodies to the pathogen in local monkeys [6–8]. Subsequently, postoutbreak in the early 2000s in central India, the CHPV garnered global attention as a human pathogen of public health importance due to its virulence and high case fatality with significant advances being made toward a basic understanding of the virus transmission patterns, pathogenicity, and in the development of diagnostics and vaccines [9]. CHPV is transmitted through the bite of infected sand flies, of which the pathogen has been isolated in both *Phlebotomus* and *Sergentomyia* spp., which serve as vectors after acquiring the virus from an unknown reservoir likely from feeding on infected humans. The yellow fever mosquito, *Aedes aegypti*, has also been implicated in the transmission of this pathogen experimentally. After ingestion, the virus replicates within the vector which subsequently can transmit the virus to new hosts [10–12].

Between June and August 2024, the Ministry of Health and Family Welfare of the Government of India reported 245 cases of acute encephalitis syndrome (AES) with a CFR of 33% (82 deaths) making this the largest outbreak in 2 decades. Of the total 245 AES cases reported, CHPV has been confirmed in 64 cases through immunoglobulin M enzyme-linked immunosorbent assay (IgM ELISA) or reverse transcription–polymerase chain reaction (RT-PCR). This resurgence of the disease is particularly concerning due to the number of recorded cases and fatalities as there is no specific treatment available for CHPV infection and management is symptomatic [13]. The transmission of this disease by sandflies means that public health measures such as insecticidal spray for vector control, information, and educational awareness, as well as adequate supportive care, have formed the mainstay of the public health response to this outbreak. Upon the institution of these measures, a declining trend of the daily reported new cases of AES has been evident since July 19, 2024 [14].

## 2. Virology and Pathogenesis

The recent emergence and resurgence of infectious diseases caused by viral, bacterial, and fungal pathogens continues to present a growing public health challenge. For example, Achromobacter xylosoxidans, a Gram-negative bacterium, is increasingly found in healthcare settings and it has demonstrated resistance to ciprofloxacin, which complicates management. Candida auris, a multi–drug-resistant fungus, is similarly causing an increase in healthcare-associated outbreaks due to its multidrug resistance [15–17]. One of the resurgent viral infections causes considerable challenges in CHPV. CHPV is a single-stranded RNA virus that has the shape of a bullet, and it measures about 150–165 nm long and 50–65 nm wide [18]. The structure of the virus consists of an envelope with a helical ribonucleoparticle (RNP) which is then surrounded by a bilayer lipid membrane in the outer layer. There is a presence of glycoprotein G which is seen in the outer membrane, while matrix protein M lies inward of the inner leaflet of the outer membrane [19]. However, the RNP is surrounded by nucleocapsid which contains viral proteins L and P that are packed in mature virion and are associated with nucleocapsid particles. There is the presence of a long protein that has a spike structure, and this aids in the adsorption, assembly, and budding of the virus [20].

The virus has an approximately 11-kb CHPV genomic RNA. Five structural proteins are encoded by the viral genome; they include nucleocapsid protein, phosphoprotein, matrix protein, glycoprotein, and large structural protein. They are manufactured in the form of five monocistronic mRNAs [18]. The nucleocapsid protein helps to initiate encapsulation of the replication product concurrent to synthesis [21], while the phosphoprotein helps in the transcription and replication of the CHPV, matrix protein is important in the assembly and budding, and this can inhibit gene expression [22]. Furthermore, the spike glycoprotein is important for the entry of the virus into the cell and is responsible for neutralizing the antibodies against the CHPV [22].

The viral life cycle involves entering host cells, primarily neuronal cells in humans, via the interaction of the viral glycoprotein G and host cell receptors such as phosphatidylserine-binding proteins or heparan sulfate proteoglycans [23]. The virus then releases genetic material, synthesizing viral proteins and progeny genome RNA, assembling mature virus particles, and exiting infected cells [23]. The virus gained entry into the cell after adsorption; this is followed up by the uncoating and release of core RNP into the cytosol from late endosomal vesicles, and the viral polymerase is responsible for the next phase of entry which is the transcription of the genome. Furthermore, the translation of viral mRNA is the next phase which is then followed by post-translational modifications of viral proteins, and the replication of the viral genome is the next step before the assembly of progeny particles and finally budding of mature virion [18].

The CHPV means of entry into the neuron and the mechanism by which it causes death is relatively unknown [24]. However, it is known to gain entry into the central nervous system by retrograde movement from peripheral nerves or olfactory nerves although it has been recorded to gain entry through hematogenous means by destroying the blood–brain barrier or infecting endothelial cells which is made possible by cytokines and chemokines produced after invading the barrier. Furthermore, the immune response to this virus entry results in the production of cellular stress factors, proinflammatory cytokines, nitrogen species, and the release of reactive oxygen species which initiate neuronal death [24].

A study conducted reported that neurons infected by the CHPV undergo apoptosis through the Fas-associated death domain (FADD) pathway, and this is possible after the activation of caspase-8 and caspase-3 and prominent cleavage of poly(ADP-ribose) polymerase (PARP) [23]. Also, it has been reported that the CHPV has been known to cause encephalitis and neuronal death [25]. Following these events, the symptoms presented are rapidly progressive due to a short incubation period ranging from 24 to 48 h. The sudden symptoms include high fever, altered sensorium, and seizures [26]. These symptoms can progress rapidly to the encephalitic syndrome; however, the disease can progress rapidly, thereby leading to acute encephalitis or swelling of the brain. Moreover, these symptoms may also lead to death within 24–72 h of the onset leading to coma and death if not adequately treated [26].

**Figure 1:**
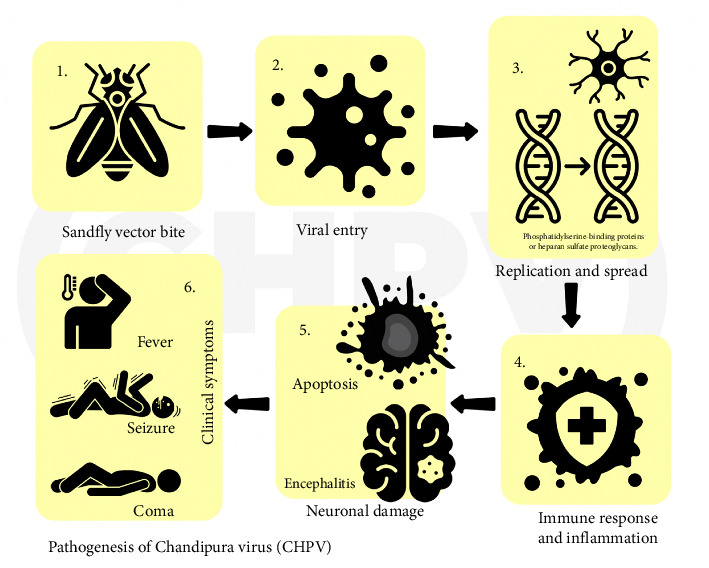
An image showing the pathogenesis of Chandipura virus.

There is a high mortality rate associated with the CHPV. As of August 2024, there has been a persistent outbreak in India, the largest in the last 20 years, where 64 confirmed cases have been reported [16].  Figure 1 illustrates the pathogenesis described previously.

## 3. Epidemiological Trends

CHPV is a disease endemic to India and a common cause of acute encephalitis in pediatric populations [12, 14]. The disease was first isolated in 1965 in Maharashtra state, India. However, retrospective studies have confirmed the first outbreaks likely occurred almost a decade prior in 1957 [12]. Following its initial discovery, the disease remained largely neglected until 2003 when an outbreak occurred in 11 districts of Andhra Pradesh resulting in 329 cases and 183 fatalities, a staggeringly high CFR of 56% [2]. The CFR of outbreaks has reached as high as 75% [13]. The disease primarily affects children under the age of 15 and displays a rapid course of progression with symptoms of high fever and vomiting typically graduating to Grade 4 coma and death within 48 h of symptom onset [12]. Fortunately, no cases of human-to-human transmission of CHPV have even been identified, it is instead transmitted by the bite of vectors such as sandflies, mosquitoes, and ticks. In particular, sandflies of the genera *Phlebotomus* and *Sergentomyia* have been directly implicated in the disease transmission, although a full understanding of the role they play remains to be reached [26]. CHPV was long thought to be isolated to India; however, entomological surveillance has revealed that the virus is present in African countries, Nigeria and Senegal, as well as Bhutan, Nepal, and Sri Lanka, on the Indian subcontinent [12]. This fact has raised concerns about the potential risk of CHPV spread to other regions, especially those with similar ecological conditions to India. Outbreaks have occurred sporadically across different Indian states and regions since 2003, and they include Andhra Pradesh (2003–2005, 2007-2008), Gujarat (2005, 2009–2012), Vidarbha region of Maharashtra (2007, 2009–2012), and Bihar (2018) However, they have largely been small and well contained [25]. The most recent outbreak of AES reported is the largest such one in 20 years—245 cases were reported between early June and August 15, 2024, and they resulted in 82 deaths, 64 of which have been confirmed to be due to CHPV. Of the 64 confirmed CHPV cases, 61 of them, 95%, have come from Gujarat State, and 3 from Rajasthan state. As reported on August 23, 2024, new cases had been decreasing daily since July 19. However, experts warn of a potential resurgence due to the ongoing monsoon season. Cases of CHPV are typically exacerbated during this season as conditions are favorable for sandfly populations and other disease vectors [13]. The situation is further complicated by factors such as urbanization, population movement, and ecological changes, which influence vector populations and increase human exposure to the virus [25]. These combined elements create a complex environment that could lead to a resurgence in CHPV cases if continued vigilance and preventive measures are not practiced.

## 4. Diagnostic Advances

Diagnosis of CHPV is usually done via epidemiologic and clinical findings followed by laboratory confirmation. However, with CHPV being endemic in low-resource countries like India, Bhutan, Nepal, Sri Lanka, Nigeria, and Senegal due to limited available resources, diagnosis in those countries has not been successfully progressive [2, 7, 13]. With the advancement in genomic diagnostics, CHPV diagnosis has been progressively advanced to ensure accuracy and precision these diagnoses include:

  RT-PCR for CHPV: Being an RNA virus, CHPV can be diagnosed using a real-time molecular diagnostic assay of one-step PCR to efficiently map the genetic makeup of CHPV [12]. Due to its sudden onset, high fatality rate and neurological complications associated with Chandipura viral encephalitis (CHPE), and serological testing alone cannot proffer the accurate result needed to efficiently diagnose CHPV [10]. RT-PCR detects as low as 10 pfu/mL from clinical specimens sufficient [12, 18]. However, advanced diagnosis is not relatively available in those communities where CHPV is endemic. Because of its 100% specificity, RT-PCR for CHPV identifies other viruses without interfering with the molecular identification of CHPV. Because of its protein functional structure at the genome level, the single-stranded RNA contains five structural proteins called monocistronic mRNAs, making RT-PCR sensitive for diagnosing CHPV in affected patients [18, 27]. As an immunological assay for serology, ELISA uses antibodies–antigen titer to measure the potency of a protein constituent capable of transmitting a disease. It is interesting to know that early serological diagnosis of CHPV did not result in anti-CHPV antibodies. This is evidence of the transient nature of this virus making serology not an effective diagnostic tool. However, sera from frogs, lizards, and rodents showed evidence of anti-CHPV in these animals as a host in the transmission of CHPV [28]. Tandale and colleagues conducted a study in Andhra Pradesh state to measure the seroprevalence of anti-CHPV neutralizing antibodies. In the pediatric population, the antibodies were discovered in 237 of 291 samples collected for investigation [29–33]. This work further strengthened the argument that pediatric age groups are susceptible and thus have CFR from CHPV [18].
  Virus Isolation for CHPV: For diagnosis purposes, CHPV has been isolated in humans and sandflies as a vector for this pathogen [34]. Other vertebrates like frogs, rodents, and bats have also been proven to abhor this virus [33]. Making laboratory investigation a significant contribution to the diagnosis of CHPV. Hence, viral culture has been a major diagnostic laboratory investigation for multiple virus isolation in the laboratory [35]. Unlike bacteria, viruses can be grown both in vivo and in vitro, artificially through test-tube, cell culture flask or agar plate to isolate or multiply this infectious virus for investigation. Primary cell culture has enabled the continuous growth of viruses including CHPV making it an invaluable instrument for virus diagnosis and research [34].
  Immunofluorescence Assay (IFA): IFA detects both antibodies and antigens specifically providing distinct identification features for the virus under investigation [36]. This diagnosis adopted an approach of fluorescence-labeled antibodies detected by colored light emitted observed under a fluorescent microscope subjected to ultraviolet light and fluorochrome filter systems. This method makes it possible for CHPV antibodies or antigens to absorb a certain amount of light efficiently to measure the wavelength adapted for CHPV based on the fluorochrome used [37].

Being a neglected tropical disease, CHPV has suffered challenges in terms of diagnosis, which has exacerbated thorough investigation of this disease, especially in low-resource countries where it is epidemic [38]. In India, CHPV exhibits a near-monsoon seasonal pattern when sandfly populations are high. This has left diagnosis slow, and more attention needs to be brought to advance diagnosis of CHPV. Also, its regional epidemic has not called the world's attention to the damage it can cause. This further expounds the reason for its neglect and bias toward global health equity. Up until the time of this research, there is no evidence of treatment or vaccine development in the pipeline; rather, there are just support care management for infected individuals and environmental control for carrier vectors [13].

## 5. Current Treatment Approaches

Currently, CHPV treatment is primarily supportive, focusing on symptom management, including fluid management, anticonvulsants, and fever control [39]. While these measures are essential, they do not prevent the onset or reduce the duration of the vesiculoviral disease. Despite recent recurring outbreaks in the central parts of India and the significant burden of causing acute encephalitis in children, no specific antiviral treatments or licensed vaccines exist. There are limited specific antiviral therapies against CHPV infection. The broad-spectrum antiviral agent ribavirin (RBV) has been shown to significantly inhibit CHPV in vitro. Using a plaque reduction assay in Vero cells, RBV exhibited 50% inhibitory concentration (IC50) at 89.84 ± 1.8 μM; it was particularly effective when administered either within 1 h after infection or 4–6 h before infection, although further clinical trials are necessary to establish its safety and efficacy in humans [40]. Similarly, favipiravir, another promising antiviral candidate, has shown activity against CHPV both in vivo and in vitro [41, 42]. Using plaque reduction assays and viral growth kinetics assays in Vero cells, favipiravir showed a half maximal effective concentration (EC50) of approximately 92.26 μM. Complete inhibition of viral replication was achieved at a concentration of 320 μM. Vero cells treated at concentrations up to 10 μg/mL showed no significant toxicity. At 100 μg/mL, 8% cytotoxicity was observed, while 50% cytotoxicity (CC50) occurred at around 172.16.100.70 22068750 μg/mL, which corresponds to a concentration of 4774 μM, resulting in a high selectivity index of 51.24 [41]. Also, in 10-day-old CD1 mice treated with 300 mg/kg/day of favipiravir, there was a 100% survival rate by the seventh day postinfection [41]. The same result was consistent in a novel C.B-17 severe combined immunodeficiency (SCID) mouse model [43]. Another study focused on using synthetic small interfering RNA (siRNA) targeting the P protein of CHPV, crucial for viral replication, and assessed the efficacy of different siRNAs (including P-2 and M-5/M-6) in vitro and in vivo [44]. In vitro experiments demonstrated that transfecting cells with P-2 siRNA significantly reduced viral titers (approximately 2 logs) when administered shortly after infection. The P-2 siRNA was particularly effective, tolerating several mismatches without losing efficacy. In the in vivo experiment, mice treated with cationic lipid-complexed P-2 siRNA showed extended survival rates after intracranial infection with CHPV. Specifically, there was a notable survival rate when infected with 100 LD50 of the virus and treated with P-2 siRNA simultaneously [44]. For infections with 10 LD50, administering two doses of siRNA resulted in a 70% survival rate. Surviving mice exhibited significantly lower brain viral loads (4 logs less CHPV titer) compared to untreated controls. Importantly, these mice did not show histopathological changes or an interferon response, indicating that the treatment effectively controlled the infection without triggering excessive immune activation. In addition to the lack of histopathological changes, parameters such as cytokine levels and immune cell activation were not significantly altered. Notably, the timing of siRNA administration was critical; administering the first dose at 2 h postinfection resulted in a higher survival rate than treatment initiated later. Despite the potential of these studies, no human trial has been carried out.

No licensed vaccine specifically targeting CHPV exists, although some vaccine candidates are in their early stages of development. The urgency for an effective vaccine is underscored by the rapid progression of the neurotrophic disease, recurring outbreaks, and lack of therapeutic options for the CHPV infection. Focusing on a recombinant glycoprotein (rGp), critical for viral entry into host cells and the ability to elicit immune responses, a study reported a significant seroconversion rate in immunized mice with high neutralizing antibody and ELISA titers of 1 : 320 and 1 : 1200, respectively [45]. This suggests that the vaccine not only stimulates an immune response but also provides protective immunity against subsequent viral infections. The antibody titers were found to be dependent on the concentration of the immunogen. Following an intracerebral challenge with a lethal dose (100 LD50) of CHPV, 90% of the immunized mice survived, demonstrating adequate protection. Also, the study explored the possibility of using this recombinant vaccine in combination with other vaccines, such as diphtheria, pertussis, and tetanus (DPT). Preliminary results indicated enhanced immune responses when combined, suggesting a feasible strategy for broader immunization efforts [45]. Same investigators in a subsequent study demonstrated that mice immunized with the combination of rGp and DPT exhibited 90% seroconversion against rGp, with high antibody titers measured at 1 : 1200 through ELISA and 1 : 320 via neutralization tests [46, 47]. These results were comparable to those observed in mice immunized solely with rGp, indicating that the DPT vaccine did not inhibit the immune response to rGp.

In addition, another study focused on developing inactivated vaccine candidates using ultracentrifugation on a 30% glycerol cushion and then subjected to differential centrifugation on a 10%–60% sucrose gradient [33]. The virus was subsequently inactivated using β-propiolactone at a dilution of 1:3500, ensuring the preservation of its immunogenic properties. The study reported neutralizing antibody titers ranging from 1 : 10 to 160 after the second dose and from 1 : 80 to 1 : 320 after the third dose, achieving 85% seroconversion after the second dose and 100% after the third [33]. Notably, all immunized mice with antibody titers above 1 : 20 survived a live virus challenge, indicating adequate protection. Interestingly, using an immunoinformatic approach, a preclinical study identified several potential B-cell and T-cell epitopes that are predicted to be highly immunogenic and nonallergenic. Among these, one candidate (referred to as V1) exhibited superior physicochemical properties, making it a promising component of the vaccine [48]. This vaccine construct has a strong binding affinity for human Toll-like receptors (TLR-3 and TLR-8), crucial players in immune response. It demonstrated stability during molecular dynamics simulations, indicating that it would likely maintain its structure and function in biological systems. Using codon optimization techniques, the multiepitope construct (MEC-CHPV) was in silico cloned into the pET28a (+) expression vector. This is particularly essential for facilitating future laboratory expression and testing of the vaccine candidate. The study is limited to in silico predictions, and further in vitro and in vivo validation is necessary to confirm the efficacy of the proposed vaccine candidate.

Similarly, another study proposed an epitope-based peptide vaccine as a strategy for CHPV prevention using an immunoinformatic approach to design a multiepitope vaccine constructed using cytotoxic T lymphocyte (CTL), helper T lymphocyte (HTL), and interferon-gamma (IFN-γ) epitopes and demonstrated stable interactions with immune receptors, specifically TLR-3 and TLR-8 [49]. The vaccine construct demonstrated high immunogenicity and nonallergenic properties, promising results that make it a suitable vaccine candidate for further development.

While promising results from antivirals and vaccine candidates are emerging, ongoing research is necessary, mainly through innovative structure-based drug design and drug repurposing, optimizing vaccine formulations, and assessing its efficacy through human clinical trials. There is also a need for collaboration among key stakeholders, including the scientific community, public health organizations, and funding agencies, to overcome the intensifying efforts to find a lasting solution.

## 6. Prevention and Control Measures

**Figure 2:**
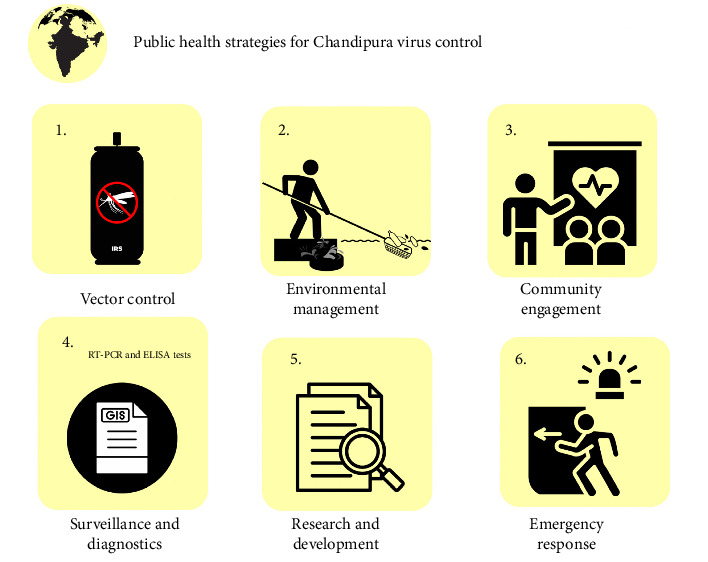
Image showing public health strategies for Chandipura virus control.

Even with the recurring nature of the CHPV outbreaks, previous experience, and most of the victims being under 15 years old, specific antiviral therapy and licensed vaccines remain elusive [50], thereby underscoring the need for effective and sustainable public health interventions, especially vector control measures to combat this vector-borne disease, especially in the rural parts of India, where vector control measures are either limited or poorly implemented. The female *Phlebotomine* sandflies, the primary vector of CHPV, thrive under warm and humid conditions, especially in the monsoon season [9]. Unsurprisingly, the ongoing outbreak coincides with the immense rainfall and flooding in India, which creates an ideal breeding ground for these vectors [50]. As such, vector control measures, mainly targeting the sandfly population, remain effective in curbing CHPV transmission, preventing further outbreaks, and ultimately, attaining an epidemic status. Another essential method of sandfly control is the use of insecticides, indoor residual spraying (IRS), and fogging, which commonly target adult sandflies. Timely application and regular monitoring ensure these measures are effective against evolving insect resistance and, consequently, the virus. Furthermore, reducing sandfly breeding sites is another crucial yet commonly overlooked measure. As climate change potentially expands the habitable zones of these vector populations, the threat of these recurring outbreaks may only increase [51, 52]; consequently, it is important to effect proper sanitation, improved drainage systems, and waste management practices, as sandflies often thrive in decaying organic matter and stagnant water. There is also a need for a joint public campaign to engage, educate, and empower local communities about proper waste disposal and environmental management. More so, it is important to monitor sandfly populations as continuous surveillance allows health authorities to adequately assess the effectiveness of current measures, identify new hotspots, and predict potential outbreaks before they even happen so that high-risk areas can receive timely and targeted interventions. Similarly, advanced technologies like geographic information systems (GIS) and remote sensing can further enhance surveillance efforts that could be leveraged to precisely map sandfly distributions and potential outbreak zones [53–55]. While these vector control measures are important, they are only as effective as their implementation. The ongoing outbreak is another reminder to ensure consistent and well-coordinated vector control strategies. There is a need for intersectoral collaboration among key stakeholders, including the scientific community, government, public health organizations, and funding agencies, to ensure these measures are made sustainable, including during the inter-outbreak period.  Figure 2 illustrates strategies employable to curbing the virus.

## 7. Public Health Implications

### 7.1. Challenges in Containment

CHPV has been circulating in India, and containment of the virus has been challenging due to several factors ranging from ecological constraints, the epidemiology of the virus, and public health system constraints [56]. Sandflies, mainly *Phlebotomus argentipes*, transmit CHPV, and they are abundant during the monsoon season when the outbreak occurs [25]. Coordinated and cheap strategies can be challenging to institute due to the seasonality of the disease and the fluctuating vector population [57]. Besides, the virus is highly prevalent in children with high case fatality rates once there is an outbreak of the disease. Many of the outbreaks have shown a mortality rate greater than 50%, and the disease advances quickly, within 24–48 h of the onset of clinical signs, thus posing challenges to early identification and management [25].

The other major challenge in preventing and controlling CHPV is the need for diagnostic procedures and effective therapies. While exciting advances such as the RT-PCR enhance the calibers of diagnostic testing, there needs to be more laboratory-supporting infrastructure where CHPV emerges in rural and isolated regions [58]. There are no official vaccines or antiviral remedies; consequently, medical intervention, consisting of treating fever, seizures, and encephalitis manifestations, remains palliative. Besides, knowledge and perception of the general population toward CHPV are low, which has led to a late presentation to the hospital, as well as a lack of adequate reporting of cases [59]. This underreporting is even worse, given the clinical signs of CHPV are like those of other encephalitis-causing pathogens, therefore making it difficult to pinpoint CHPV-specific outbreaks.

Finally, the social and geographical restraints, specifically in the Indian area, are a CHPV regulation problem. These outbreaks have occurred in various states, including Andhra Pradesh, Maharashtra, and Gujarat, regions characterized by different ecological conditions that make it difficult to have single strategies for dealing with the diseases [25]. Rural populations suffer the most from the diseases due to poor access to health facilities, surveillance, and vector management. Additionally, increased human encroachment into urban areas and changes in climate and environment could increase the human-sandfly interactions, raising the risk of CHPV transmission. These, exacerbated by weak public health systems, resulted in high outbreaks and underlined the need for enhanced politico-legal measures on surveillance, vector control, and health enhancement crusades.

### 7.2. Potential for Future Outbreaks

CHPV presently does not pose a serious threat to the people of India. However, its exposure in the future remains alarming because of its potential to cause severe encephalitis, especially in children [60]. The epidemics are seasonal occurring between May and September and most recently re-emerged in the endemic states of Gujarat, Andhra Pradesh, and Maharashtra. Since CHPV is still classified as an “emerging *viral hemorrhagic fever*,” there are currently no vaccines nor environmental therapies for this disease, thus making it highly risky to society [41]. The case fatality rate lies between 28% and 78%; therefore, timely diagnosis is essential. The rapid resurgence of cases in those states shows ample room for better sentinel surveillance and infection prevention and control (IPC) endeavors.

Additional factors such as environmental and demographic aspects continue to pose the risk of future outbreaks of CHPV. Large population density and comparatively high rate of urbanization in India also facilitate viral transmission as the sandfly breeds in and around domestic habitats [61]. Pigs, cattle, and sheep can be potential carriers of the virus, which enhances the chances of the virus getting into the human population. Like most RNA viruses, this virus exhibits certain genetic variability which enables the virus to evolve [62]. This ability to change is beneficial when the virus is introduced to new hosts and conditions, enabling it to adapt and survive more effectively [62]. The distribution patterns of the disease show that the most affected age group is the vulnerable group that is below the age of 15 years and has a high risk of severe conditions such as fever, unconsciousness, and seizures leading to death within 2 days. The fact that there is no well-established and easily accessible diagnostic test for CHPV also poses an increased risk to future occurrences. Among these challenges, lack of early diagnosis and inadequate or poorly controlled follow-up research are major obstacles to the virus [63]. Epidemiological studies have attributed the virus to sandflies, but there is still some deficit in defining the mode of transmission that fully involves these vectors. The use of surveillance, in addition to other vector control mechanisms, remains important in containing the virus [63]. With regard to patients, early diagnosis, and the presence of CHPV, awareness and preventive measures shall minimize the hurdles that future CHPV outbreaks in India.

### 7.3. Research Gaps

Even now, many future research areas have been discovered in the research of CHPV, particularly regarding the pathogenicity of the virus and the immunological control. Though CHPV has existed for over half a century, its capacity as a pathogen has not been fully uncovered [64]. The research has mostly addressed neurological consequences of the virus; however, studies should be carried out to document the survivors' long-term outcomes, specifically children in whom the virus may have left a long-term effect on cognition development [65]. Moreover, no viral progression and immune system interaction is quite clear, and further investigation is required to establish whether some sections of the population are more susceptible to severe consequences. Another significant research deficit is identifying treatments or vaccinations for the CHPV. Currently, no antiviral drugs for the virus exist, and treatment calls are being made to sustain patient care [66]. The absence of specific medications greatly slows down the strategies to decrease mortality rates. To date, there is no preventive vaccine against the CHPV; however, there have been advancements in the research and development of the vaccine in recent years [49]. Creating a vaccine is not easy, partly because of the virus and the capacity to conduct trials in areas where the cases are rare and difficult to predict. It is necessary to eliminate all the gaps to reduce people's future health threats due to the outbreak of the CHPV in India.  Table 1 summarizes the key aspects of the manuscript, while Table 2 summarizes key studies on the CHPV.

## 8. Conclusion

In conclusion, the resurgence of CHPV continues to pose significant public health challenges, particularly in the pediatric population, with its rapid progression and high fatality rates. The 2024 outbreak in India underscores the virus's persistent threat, demonstrating the need for improved diagnostic tools, timely public health interventions, and vector control measures. The review of current literature highlights the substantial progress made in understanding the virology, transmission, and pathogenesis of CHPV, as well as advancements in diagnostic techniques like RT-PCR and ELISA. However, gaps remain in terms of effective treatment and vaccine development, as the current management is limited to supportive care, with experimental antiviral therapies such as RBV and favipiravir showing promise in preliminary studies. Future research should focus on advancing antiviral treatments, accelerating vaccine development, and enhancing surveillance systems, especially in resource-limited settings where outbreaks are most frequent. The need for international collaboration to strengthen vector control measures and improve public awareness cannot be overstated, particularly as environmental and demographic factors continue to contribute to the spread of the virus. Comprehensive efforts combining research, public health policies, and global cooperation are crucial to mitigating the impact of CHPV and preventing future outbreaks.

## Figures and Tables

**Table 1:** Summary of key insights, current challenges, and strategic priorities for addressing Chandipura virus (CHPV).

Section	Key points	Current challenges
Pathogenesis	CHPV infects humans via sandfly bites, enters neuronal cells, and triggers immune responses causing neuronal apoptosis, leading to rapid encephalitis and death.	Limited understanding of exact neuronal entry and immune evasion mechanisms.
Epidemiology	Primarily affects children under 15 in India with outbreaks linked to monsoon seasons, also detected in West Africa and Sri Lanka.	Potential geographic expansion due to climate change and urbanization.
Diagnostics	RT-PCR and ELISA are key diagnostic tools; limited accessibility in low-resource settings hinders early detection.	High cost and complexity of diagnostics in rural and resource-limited areas.
Treatment	No specific antiviral treatment; supportive care is standard. Ribavirin and favipiravir show promise in preclinical studies.	Lack of clinical trials for promising antiviral drugs.
Prevention and control	Vector control (insecticide spraying, sanitation), public health education, and surveillance are crucial for outbreak prevention.	Sustaining consistent vector control and community engagement is challenging.
Public health impact	High mortality rates and rapid disease progression challenge containment; poor diagnostics and weak health systems worsen outbreaks.	Poor surveillance, underreporting, and limited public awareness in rural areas.
Research gaps	Urgent need for vaccine development, antiviral therapies, and enhanced diagnostic tools. Research is needed on virus pathogenesis and long-term outcomes.	Limited funding and resources dedicated to CHPV-specific research.

**Table 2:** Summary of evidence.

Study	Year	Methodology	Key findings	Relevance to CHPV
Chadha et al., American Journal of Tropical Medicine and Hygiene [3]	2005	Epidemiological investigation; outbreak analysis in Gujarat, India	Characterized a significant CHPV outbreak with elevated pediatric mortality rates	Established CHPV as a major etiological agent of acute encephalitis syndrome (AES) in India, necessitating enhanced surveillance
Dwibedi et al., National Medical Journal of India [5]	2015	Epidemiological study in Odisha, India	Identified CHPV among tribal populations, emphasizing regional susceptibility	Expanded the geographic and demographic understanding of CHPV transmission risk factors
Ghosh and Basu, National Medical Journal of India [24]	2017	Neuropathogenesis study	Demonstrated CHPV-induced oxidative stress, cytokine dysregulation, and neuronal apoptosis	Expanded the geographic and demographic understanding of CHPV transmission risk factors
Kumar et al., BMC Infectious Diseases [27]	2008	Diagnostic study; real-time RT-PCR assay	Developed a highly specific one-step RT-PCR assay for CHPV detection	Enhanced diagnostic accuracy and outbreak response capabilities
Tandale et al., Journal of Medical Virology [29]	2008	Epidemiological study; Andhra Pradesh outbreak	Identified CHPV in 51% of AES cases, reporting a 56% case fatality rate	Strengthened evidence implicating CHPV as a leading cause of pediatric AES
Damle et al., Japanese Journal of Infectious Diseases [31]	2018	Vector surveillance study	Confirmed CHPV presence in *Sergentomyia* sandflies in Gujarat	Reinforced the role of sandflies as primary CHPV vectors, shaping vector control strategies
Cherian et al., PLoS One [32]	2012	Genomic sequencing study	Conducted whole-genome sequencing and phylogenetic comparisons of CHPV isolates	Advanced molecular epidemiology and evolutionary understanding of CHPV
Shanmugaraj, Journal of Zoonotic Diseases [38]	2024	Epidemiological and clinical review	Examined recent CHPV outbreaks and evolving clinical presentations	Provided contemporary epidemiological data critical for public health planning
Balakrishnan and Mun, Journal of Medical Virology [40]	2020	In vitro antiviral study	Demonstrated that ribavirin inhibits CHPV replication with an IC50 of 89.84 µM	Suggested ribavirin as a potential antiviral, necessitating further clinical investigation
Pavitrakar and Bondre, Journal of Medical Virology [41]	2023	In vivo antiviral study in murine models	Favipiravir (300 mg/kg/day) conferred 100% survival in CHPV-infected mice	Provided robust preclinical evidence supporting favipiravir as a candidate antiviral
Cain and Ly, Journal of Medical Virology [42]	2023	In vitro and in vivo antiviral evaluation	Investigated favipiravir's efficacy in inhibiting CHPV replication and disease progression	Reinforced the therapeutic potential of favipiravir for CHPV management
Kitaura et al., Antiviral Research [43]	2023	SCID mouse model study	Developed novel preclinical models for CHPV antiviral evaluation	Enhanced translational research pathways for CHPV antiviral testing
Banik et al., Microbial Pathogenesis [48]	2022	Immunoinformatics-based vaccine design	Developed a multiepitope peptide vaccine targeting B- and T-cell responses	Provided a computational framework for rational CHPV vaccine design
Kanabar et al., Cureus [25]	2024	Systematic review of CHPV outbreaks	Conducted a meta-analysis on CHPV outbreak trends across India	Informed predictive modeling and preparedness strategies for future outbreaks
Salomón et al., WHO Report [58]	2022	Global vector control study	Outlined comprehensive international strategies for sandfly vector control	Established guidelines for CHPV vector management and outbreak mitigation
Jancarova et al., Journal of General Virology [61]	2023	Sandfly vector ecology study	Investigated ecological determinants of sandfly-mediated CHPV transmission	Expanded vector ecology knowledge relevant to CHPV risk assessment

## Data Availability

Data and materials were not used for this study.
